# Leveraging Machine Learning to Predict Warfarin Sensitivity in the Puerto Rican Population: A Pharmacogenomic Approach

**DOI:** 10.3390/ijerph23030337

**Published:** 2026-03-07

**Authors:** Jorge E. Martínez-Jiménez, Yolianne Ortega-Lampón, Dylan Cedres-Rivera, Frances Heredia-Negrón, Abiel Roche-Lima, Jorge Duconge

**Affiliations:** 1 Department of Biochemistry, School of Medicine, Medical Sciences Campus, University of Puerto Rico, San Juan, PR 00936, USA; jorge.martinez20@upr.edu; 2 Department of Chemistry, Rio Piedras Campus, University of Puerto Rico, San Juan, PR 00925, USA; 3 Integrated Informatics Services Core (IIS), Research Center in Minority Institution (RCMI), Medical Sciences Campus, University of Puerto Rico, San Juan, PR 00936, USAfrances.heredia@upr.edu (F.H.-N.); 4 Department of Pharmacy Practice, School of Pharmacy, Medical Sciences Campus, University of Puerto Rico, San Juan, PR 00936, USA

**Keywords:** machine learning, pharmacogenomics, ethno-specific variants, drug response prediction

## Abstract

**Highlights:**

**Public health relevance—How does this work relate to a public health issue?**

- Warfarin therapy is associated with substantial adverse drug events and hospitalizations in older adults.
- Genetic and clinical heterogeneity complicates safe warfarin dosing in admixed Hispanic populations.

**Public health significance—Why is this work of significance to public health?**

- Many existing warfarin pharmacogenomic models have limited applicability across populations with differing genetic architectures.
- Incorporation of population specific genetic variation improves classification of warfarin sensitivity.

**Public health implications—What are the key implications or messages for practitioners, policymakers, and/or researchers?**

- Population-informed prediction models may improve the clinical management of anticoagulation therapy.
- Broader representation of genetic backgrounds is needed to enhance the generalizability of pharmacogenomic tools.

**Abstract:**

Warfarin is one of the most used oral anticoagulants, even after the arrival of non-vitamin K oral anticoagulants. Warfarin has been implicated in approximately one-third of emergency hospitalizations for adverse drug events among older adults in national U.S. data. Warfarin dose has been shown to vary between patients with up to 10 times the standard dose. This variability is due to multiple factors such as age, gender, diet, body size, co-medications, and the genetic background of the patient, where the genetic background accounts for 50% of warfarin dose variability among Europeans. Sadly, these findings do not apply to Caribbean Hispanic populations such as Puerto Ricans due to them having an admixed genetic profile. In the field of pharmacogenomics (PGx), the utility of machine learning (ML) has been used to predict individual drug responses by analyzing complex genetic and clinical data, which helps personalize medicine by tailoring treatments to a patient’s genetic makeup. Inclusion of ethno-specific variants has demonstrated improvement on the application of ML to a specific population. This study compares eight ML methods to predict warfarin sensitivity in Puerto Rican Caribbean Hispanics. This study is a secondary analysis of genetic and clinical data from 217 Puerto Rican patients treated with warfarin for thromboembolic disorders. After quality control filtering and exclusion of participant records with incomplete genetic and clinical data, 146 participants are retained for analysis. Data are divided into 65% and 35% to be used as training and test sets. Model performance is determined by comparing the precision and accuracy metrics, computed through the corresponding confusion matrixes. A gradient boosting classifier (GDB) achieves the highest overall accuracy (0.7500) and weighted precision of (0.7642); however, sensitivity for detecting warfarin-sensitive patients remains low. Feature importance analysis suggests that rs202201137 could contribute to model predictions, although overall detection of warfarin-sensitive individuals remains limited.

## 1. Introduction

Pharmacogenomics (PGx) is the interdisciplinary field that integrates pharmacology, genomics, and molecular biology to understand how genetic differences affect an individual’s response to drugs. It focuses on identifying genetic markers, such as single nucleotide polymorphisms (SNPs), copy number variations, and haplotypes, that influence drug efficacy, toxicity, and dosage requirements [1]. The term “pharmacogenetics” was first introduced in the 1950s, when researchers discovered that specific genetic variations could lead to adverse drug reactions, such as the sensitivity to antimalarial drugs in individuals with glucose-6-phosphate dehydrogenase deficiency [2]. By using genetic information, PGx aims to develop tailored treatments, reduce adverse drug events, and enhance therapeutic outcomes, marking a paradigm shift from one-size-fits-all to personalized medicine [3]. Clinically, it has revolutionized drug therapy by enabling precise dosing and treatment regimens based on genetic profiles, minimizing trial-and-error prescribing [4]. For instance, PGx insights have been integrated into clinical guidelines for drugs such as warfarin, clopidogrel, and antidepressants, improving patient safety and treatment success [5,6,7,8]. Furthermore, PGx contributes to addressing health disparities by studying genetic variations in diverse populations, paving the way for equitable healthcare advancements and personalized interventions for underrepresented groups [9].

Warfarin, introduced as an anticoagulant in the 1950s, became one of the most widely prescribed agents for the prevention and treatment of thromboembolic disorders, including venous thromboembolism and atrial fibrillation [10]. Despite its clinical efficacy, warfarin has a narrow therapeutic index (NTI), meaning there is a small margin between its effective and toxic doses [11,12]. Even minor changes in dose, drug interactions, diet, or patient-specific factors could result in serious bleeding complications. Consequently, NTI drugs such as warfarin require careful dose titration and close monitoring to ensure safe and effective therapy. National U.S. surveillance data have shown that warfarin is implicated in approximately 33% of emergency hospitalizations for adverse drug events among adults aged ≥65 years between 2007 and 2009 [13]. These findings underscore the substantial clinical and public health burden associated with warfarin therapy. Early pharmacogenetic studies in the 1990s identified genetic variation in the cytochrome P450 family 2 subfamily C member 9 (*CYP2C9*), cytochrome P450 family 4 subfamily F member 2 (*CYP4F2*), and vitamin K epoxide reductase complex subunit 1 (*VKORC1*) genes as major determinants of interindividual variability in warfarin metabolism and dose requirements [14,15,16,17]. Incorporation of these pharmacogenetic (PGx) markers into dosing algorithms has been shown to reduce the time required to achieve therapeutic anticoagulation and may decrease the risk of adverse events in some populations [18].

Current clinical implementation of pharmacogenetic-guided warfarin dosing is informed primarily by recommendations from the Clinical Pharmacogenetics Implementation Consortium (CPIC). The 2017 CPIC update incorporates genetic variation in *CYP2C9*, *VKORC1*, *CYP4F2*, and the *CYP2C* cluster variant rs12777823 to guide warfarin dose selection in adults with a target international normalized ratio (INR) of 2–3. Common variants in VKORC1*2 (rs9923231), *CYP2C9* (e.g., CYP2C9*2 [rs1799853] and CYP2C9*3 [rs1057910]), and CYP4F2*3 (rs2108622) account for a substantial proportion of stable dose variability, approximately 30%, 18%, and 11%, respectively, in European ancestry populations [15]. CPIC recommends the use of validated pharmacogenetic dosing algorithms that integrate genotype with clinical variables such as age, body size, target INR, and interacting medications to estimate therapeutic dose requirements. Importantly, the 2017 update emphasizes ancestry specific considerations. In individuals of African ancestry, decreased function *CYP2C9* alleles (*5, *6, *8, *11) and rs12777823 significantly influence dose requirements, with recommended dose reductions of approximately 15–30% per *CYP2C9* variant allele and 10–25% in rs12777823 A allele carriers. These updates represent meaningful progress toward improving equity in pharmacogenetic implementation by recognizing population-specific genetic architecture.

Nevertheless, despite these advances, substantial gaps remain for Hispanic/Latino populations, particularly admixed Caribbean groups. Most pharmacogenomic studies, including genome-wide association studies (GWAS), have been conducted predominantly in individuals of European ancestry, [9,19,20,21] with more than 87% of participants in many genomic datasets of European descent and fewer than 2% Hispanic/Latino individuals [22]. As a result, dosing algorithms derived primarily from European ancestry cohorts may not fully capture genetic determinants of the drug response in admixed populations, potentially contributing to suboptimal dosing recommendations and an increased risk of adverse drug events [23]. A review by Popejoy reported that, among 102 North American genomic studies, only six focused exclusively on Hispanic/Latino populations [23]. Puerto Rican populations are particularly underrepresented despite their unique tri-hybrid admixture of African, Native American Taíno, and European ancestry [24]. Studies tailored to genetically admixed populations, such as Puerto Ricans, are necessary to refine dosing frameworks and ensure that advances in PGx benefit all patient groups [9,19,20,21,23,24].

Machine learning (ML) is a subset of artificial intelligence that enables computer systems to learn and improve from experience without being explicitly programmed. It involves the development of algorithms that can analyze data, recognize patterns, and make predictions or decisions based on input data [25]. It has emerged as a powerful tool in PGx, processing complex genetic data to predict drug responses and enable personalized treatments [26]. By training on labeled genomic datasets, these algorithms can predict outcomes, classify genetic variants, and identify key features contributing to observed traits, enabling more precise interpretations of genetic information [27]. ML algorithms are used to identify SNPs and genetic markers associated with diseases through GWAS [28]. Application of ML has also been associated with enhanced development of polygenic risk scores by integration from multiple SNPs and utilization of nonlinear relationships and higher order interactions between variants [29,30]. Additionally, ML has been impactful on functional genomics, helping interpret non-coding regions of the genome by predicting the regulatory impact of variants, as well as evolutionary studies by reconstructing population histories, inferring migration patterns, and analyzing haplotype structures, providing insights into human evolution and the genetic basis of population-specific traits [31]. ML also facilitates feature selection, a critical step in reducing the dimensionality of genetic data while retaining its most informative aspects [32]. We aim to integrate machine learning with Puerto Rican-specific *CYP2C9* variants rs2860905 and rs202201137 that have been associated with lower warfarin dose requirements to develop and evaluate exploratory predictive models of warfarin sensitivity, improving dosing accuracy and clinical outcomes in the Puerto Rican population [32].

## 2. Materials and Methods

Patient Cohorts—This was a secondary analysis of genetic data and statin use collected from 217 patients from patients on warfarin who were recruited earlier at medical facilities in the VA Caribbean Healthcare System, San Juan, P.R.; the University of Puerto Rico’s Hospital Dr. Federico Trilla, Carolina, P.R.; the Miami VA Healthcare System, Miami, Fl.; and the Brownstone Anticoagulation Clinic of Hartford Hospital, Hartford, CT. (protocol #2290034576). The protocol number (#2290034576) corresponds to IRB approval from the University of Puerto Rico Medical Sciences Campus. The original informed consent included broad consent provisions permitting future secondary analyses of genetic and clinical data. Participants of the study were using warfarin (main inclusion criteria). In addition, enrolled volunteers satisfied all the following criteria to be eligible for the study: Caribbean Hispanic ethnicity; aged 21 and above; patients stable on warfarin (i.e., at least three consecutive INR measures on target for the same average weekly dose); duration on warfarin therapy of at least 3 months; availability of non-genetical data (i.e., age, gender, body weight, warfarin doses, INRs, co-meds, etc.); and willingness and ability to sign informed consent. This clinical study was conducted according to the principles in the Declaration of Helsinki. Written informed consent was obtained from each participant before enrollment. From these 217 patients, only 146 patients had complete genetic and clinical information to be used for future analysis.

Variant Selection—The selection of genetic variants was guided by prior evidence of clinical relevance in warfarin dose variability. The Puerto Rican-specific CYP2C9 variants rs202201137 and rs2860905 were previously identified in candidate gene association studies conducted in Caribbean Hispanic patients receiving warfarin therapy where they were associated with lower dose requirements [33,34,35]. Additional variants including VKORC1*2, CYP2C9*2/*3/*5/*6/*8/*11, CYP4F2*3, and NAD(P)H quinone oxidoreductase 1 (*NQO1*)*2 (rs1800566) were selected based on their established role in warfarin pharmacokinetics and pharmacodynamics, and their inclusion in CPIC genotype-guided dosing recommendations [5]. Variants such as *CYP2C9* rs1856908, *ABCB1* rs10276036, and *CES2* rs4783745 were previously evaluated in admixed Latino populations and demonstrated associations with warfarin dose variability in Caribbean Hispanic cohorts [34].

Dataset Preparation—The analytical dataset was constructed by integrating pharmacogenetic variants and clinical covariates relevant to warfarin dose variability. Genetic predictors included Puerto Rican ethno-specific *CYP2C9* variants (rs2860905 and rs202201137), as well as established pharmacogenetic markers associated with warfarin dose requirements, including CYP2C9*2/*3/*5/*6/*11, NQO1*2, VKORC1*2, CYP2C9*8 (rs7900194), and additional loci (Table 1). Clinical variables included age, sex, body mass index (BMI), and height. A complete list of variables is provided in Table 1.

All features were encoded numerically for model development. Genotypes were coded as 0, 1, or 2 according to the allele count, and missing values were initially coded as −9. Prior to preprocessing, −9 values were recoded as missing (NaN) to ensure compatibility with downstream modeling procedures.

Missing genotype entries were observed for a subset of pharmacogenetic variants, including rs202201137 (*n* = 3); rs2860905 (*n* = 1); rs1856908 (*n* = 68); *ABCB1* rs10276036 (*n* = 68); *CES2* rs4783745 (*n* = 68); CYP4F2*3 (*n* = 50); and NQO1*2 (*n* = 49). Model-based prediction using a random forest classifier (RFC) was applied exclusively to the genotype categories (0/1/2 coding) and did not involve reference-panel-based imputation or haplotype reconstruction. This method enabled modeling of nonlinear relationships and interactions while minimizing parametric assumptions [36,37].

**Figure 1:**
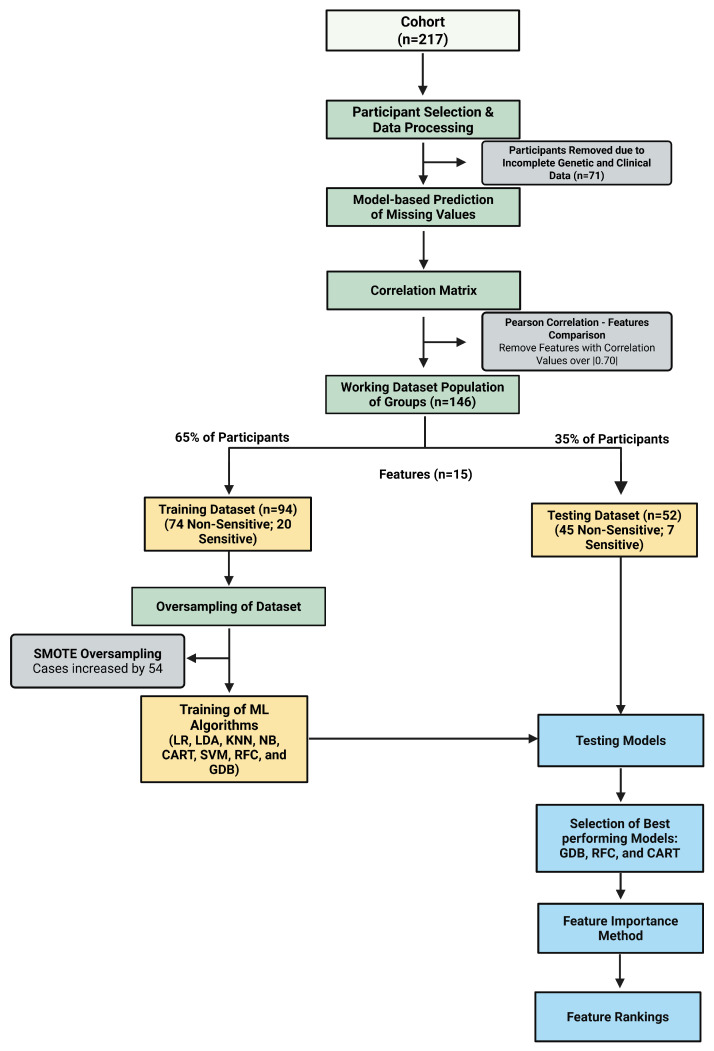
Flowchart describing methodology from dataset preparation to permutation of feature importance. In summary, initial cohort of 217 patients was processed for analysis, and 71 patients were removed due to mismatching features. Model-based predicting of missing values and oversampling after splitting was performed to have a more balanced dataset. Correlation matrix was used to remove features with correlation values over |0.70|. Dataset was divided into 65% for training and 35% for testing. Linear discriminant analysis (LDA), gradient boosting classifier (GDB), decision tree classifier (CART), support vector machine classifier (SVM), logistic regression (LR), random forest classifier (RFC), k-nearest neighbor (KNN), and Gaussian naïve Bayes (NB) were tested and tuned in order to select the best performing model. For the best performing models (GDB, RFC, and CART), feature importance rankings were determined using the permutation_importance function implemented in scikit-learn. Figure was created in Biorender [44].

Patients were classified into two groups based on stable weekly warfarin dose requirements: sensitive (≤21 mg/week) and non-sensitive (>21 mg/week). This threshold corresponded to approximately 3 mg/day, a commonly used cut-point to identify patients requiring lower-than-average maintenance doses in pharmacogenetic studies of warfarin dosing [38]. Patients requiring ≤21 mg/week were generally considered to have increased warfarin sensitivity due to a reduced metabolic capacity or an enhanced pharmacodynamic response, frequently associated with variants of *CYP2C9* and *VKORC1* [39,40,41]. Similar thresholds have been applied in prior pharmacogenetic analyses evaluating warfarin dose variability [42]. Among the 146 patients included in the final dataset, 27 were classified as sensitive and 119 as non-sensitive. The dataset was divided into 65% training and 35% validation sets. Due to the class imbalance, synthetic minority oversampling (SMOTE) was applied to the training data to balance the sensitive and non-sensitive groups prior to model development [43]. All preprocessing steps were performed within the training framework to avoid data leakage. The overall methodological workflow is illustrated in Figure 1.

Computational Environment—All analyses were conducted in Python 3.11.14 (MSC v.1929, 64-bit) on a Windows 10 platform. Machine learning models were implemented using scikit-learn 1.7.2, with data manipulation performed using pandas 2.3.3 and NumPy 1.26.4. Class imbalance handling was conducted using imbalanced-learn 0.14.0. Model interpretability analyses were performed using SHAP 0.49.1. Figures were generated using Matplotlib 3.10.6.

Machine Learning Algorithms—Eight supervised learning classification algorithms were selected for model generation and testing. These algorithms were linear discriminant analysis (LDA), gradient boosting classifier (GDB), decision tree classifier (CART), support vector machine classifier (SVM), logistic regression (LR), RFC, k-nearest neighbor (KNN), and Gaussian naïve Bayes (NB). LDA is a dimensionality reduction technique that aims to project data onto a lower-dimensional space while maximizing the separation between multiple classes, improving classification performance [45]. GDB fits new models to provide a more accurate estimate of the response variable to construct the new base learners, typically decision trees, sequentially to be maximally correlated with the negative gradient of the loss function associated with the whole ensemble [46]. CART is a supervised machine learning tool that splits data into branches based on feature values, creating a tree-like model of decisions to classify data points that are filtered down through the tree to get the correct output [47]. SVM finds the optimal hyperplane to separate data points of different classes with the maximum margin, working well in high-dimensional spaces when the number of dimensions exceeds the number of samples [48]. LR is used for binary classification tasks that models the probability of a categorical dependent variable using a logistic function, predicting the likelihood of an event by fitting data to a logistic curve, providing outputs between 0 and 1 [49]. RFC is a classification algorithm that builds multiple decision trees during training and merges their outputs to improve the accuracy and control overfitting, using the averages of results from many trees to provide more reliable predictions compared to individual decision trees [50]. KNN classifies a data point based on the majority class among its k-nearest neighbors in the feature space, making predictions by calculating the distance between the data points and assigning the most common class among the nearest ones [51]. Lastly, NB is based on Bayes theorem, which is a probabilistic classifier that assumes the features follow a Gaussian distribution and are conditionally independent given the class label, outputting the probability of class membership by considering all features in unison [52].

Model Performance Evaluation—These ML algorithms were trained using the “training set” in two formats (raw data and scaled data). Scaled data was computed using the StandardScaler function from scikit-learn to standardize features by removing their mean and scaling to unit variance, facilitating improved performance for certain machine learning algorithms [53]. Model performance was evaluated using both accuracy and precision in predicting warfarin dosing classification. The best ML-based models were selected using precision and accuracy metrics, computed through the corresponding confusion matrixes [54]. Accuracy was calculated using the following formula, $\frac{True\;Negatives+True\;Positives}{True\;Negatives+True\;Positives+False\;Positives+False\;Negatives}$, across the 10 folds, and its corresponding standard deviation were used to compare model performance. Both unscaled and scaled versions of each algorithm were assessed, with scaling applied through standardized pipelines to prevent data leakage. Precision was calculated with the following formula, $\frac{True\;Positives}{True\;Positives+False\;Positives}$, using the same cross-validation structure but substituting the scoring parameter. The final model performance was evaluated on an independent validation cohort that was not used during training or hyperparameter tuning. Classification performance was quantified using multiple complementary metrics derived from the confusion matrix: accuracy, recall (sensitivity), specificity, positive predictive value, negative predictive value, and weighted precision. Sensitivity and specificity quantified minority class and majority class discrimination, respectively. Weighted precision was obtained from the classification report and reflected class-specific precision values weighted by class support, thereby accounting for the class imbalance. Given the imbalance between warfarin-sensitive and non-sensitive individuals, precision–recall performance was additionally evaluated using the area under the precision–recall curve (PR-AUC). PR-AUC was computed from predicted class probabilities on the validation set. To estimate uncertainty in precision–recall performance, 95% confidence intervals for PR-AUC were generated using non-parametric bootstrapping (2000 resamples). For each bootstrap iteration, validation samples were resampled with replacements and PR-AUC was recalculated; percentile-based confidence bounds were then derived from the empirical distribution. Feature importance was evaluated using two approaches to quantify the contribution of each predictor. First, impurity-based feature importance was obtained directly from tree-based classifiers, including CART, RFC, and GDB models. These methods calculate importance as the total reduction in node impurity attributable to each feature across all splits in the model [55]. Features producing larger reductions in impurity were assigned higher importance scores. Impurity-based importance values were extracted using the feature_importances attribute of each fitted model. Second, model agnostic permutation importance was computed to assess the impact of each feature on predictive performance independent of the model’s internal structure. For each predictor, values were randomly permuted while holding all other features constant, and the resulting decrease in classification accuracy was measured. A larger drop in accuracy indicated a greater contribution of that feature to model performance. Permutation importance was implemented using scikit-learn’s permutation_importance function with multiple shuffling iterations to obtain mean importance scores and associated variance.

## 3. Results

### 3.1. Predictive Algorithm Comparison

**Figure 2:**
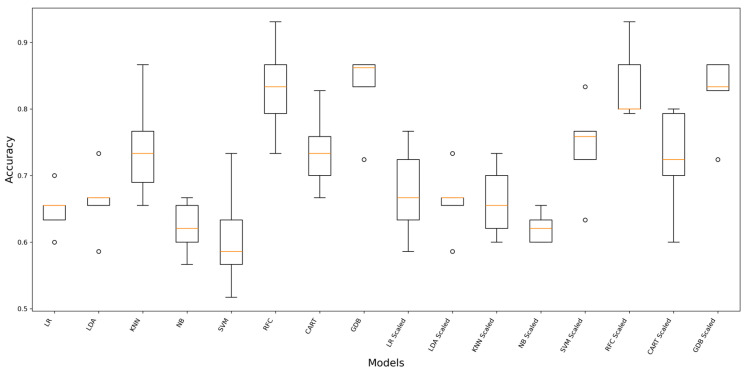
Boxplot for warfarin classification performance of ML models. Boxes represent the interquartile range with the median shown as the central line; whiskers extend to 1.5 times the interquartile range, and hollow circles indicate outlier values.

Figure 2 presents the cross-validated accuracy distributions for each algorithm under scaled and unscaled preprocessing. Ensemble tree-based models (GDB and RFC) achieved the highest and most stable performance overall, with median accuracy around 0.85 and 0.83, respectively, and relatively narrow interquartile ranges. CART displayed moderate performance with wider dispersion, while SVM and NB exhibited lower median accuracies and greater variability. KNN showed intermediate performance, outperforming SVM and NB, but remained below the ensemble methods. Feature scaling substantially influenced certain algorithms. The most pronounced improvement was observed for SVM, where the scaled implementation demonstrated a marked increase in median accuracy (approximately mid 0.70 s) and reduced variability compared with its unscaled counterpart.

### 3.2. Predictive Algorithm Tuning

Table 2 summarizes class-specific performance metrics for the three best performing machine learning algorithms evaluated on the independent validation set. Overall accuracy ranged from 0.65 to 0.75, with GDB achieving the highest accuracy (0.7500), followed by RFC (0.7308) and CART (0.6538). Weighted precision ranged from 0.7176 to 0.7642, again with highest for GDB. Specificity for identifying non-sensitive patients was consistently moderate to high, ranging from 0.7556 (CART) to 0.8444 (GDB). In contrast, sensitivity for identifying warfarin-sensitive patients remained low across the models (0.1429 for GDB and RFC, and 0 for CART), reflecting limited detection of the minority class. Positive predictive value (PPV) was similarly low (0–0.125), whereas negative predictive value (NPV) remained comparatively high (0.8293–0.8636), consistent with the class imbalance in the dataset. Precision–recall performance was modest, with PR-AUC values ranging from 0.1346 to 0.1786. The highest PR-AUC was observed for the random forest classifier (0.1786; 95% CI: 0.068–0.442), followed closely by gradient boosting (0.1686; 95% CI: 0.066–0.419). Confidence intervals overlapped across models, indicating no statistically meaningful separation in precision–recall performance.

### 3.3. Permutation Feature Importance

As shown in Table 3, feature importance varied across algorithms, although several predictors demonstrated consistent relevance. *CYP2C9* rs202201137 (A > G) ranked among the most influential genetic variants overall (mean rank = 4.67), maintaining moderate importance across CART, GDB, and RFC. VKORC1*2 also demonstrated consistent contribution (mean rank = 5.0), ranking within the top six features in both GDB and RFC. Among clinical variables, BMI and height in inches showed strong but model-dependent effects (mean rank = 5.33 for both). BMI ranked first in CART and second in GDB but was substantially lower in RFC, whereas height ranked first in GDB and second in RFC but was less influential in CART. These findings suggest that clinical variables contributed meaningfully to predictive performance, although their relative importance varied by algorithm. Among the ethno-specific variants, CYP2C9 rs202201137 (A > G) demonstrated the most consistent overall influence (mean rank = 4.67), maintaining moderate importance across CART, GDB, and RFC, whereas CYP2C9 rs2860905 (G > A) showed greater variability across algorithms (mean rank = 6.0), ranking higher in CART and RFC but contributing less prominently in GDB.

## 4. Discussion

Using scaled and non-scaled datasets, we found different performances from the eight ML algorithm used to predict warfarin sensitivity in our small cohort of Puerto Rican patients. Because no universally optimal scaling strategy existed for all algorithms [56], models were trained and evaluated using both raw and standardized feature representations. However, after hyperparameter tuning and independent validation, ensemble tree-based methods demonstrated the strongest overall performance. GDB achieved the highest validation accuracy (0.75), followed by RFC (0.73), while CART showed lower discrimination (0.65) (Table 2). Evaluation of class-specific metrics revealed important limitations. Sensitivity for identifying warfarin-sensitive individuals was low (0.14 for GDB and RFC; 0.00 for CART), whereas specificity for identifying non-sensitive individuals ranged from 0.76 to 0.84. Positive predictive value was similarly low (0–0.125), while negative predictive value remained high (0.83–0.86), reflecting the class imbalance within the validation cohort. Precision–recall performance was modest (PR-AUC 0.13–0.18), with overlapping confidence intervals across models, indicating limited discriminatory separation. Collectively, these findings suggest that although ensemble methods demonstrated relatively stable performance, discrimination of the minority (warfarin-sensitive) class remained constrained in this small validation sample. Rather than demonstrating immediate clinical readiness, these results should be interpreted as evidence of feasibility, as predictive frameworks incorporating population-specific pharmacogenetic variants can be constructed and evaluated in admixed Caribbean cohorts, but larger sample sizes will be required to achieve clinically meaningful sensitivity.

Permutation-based feature importance analysis revealed heterogeneous ranking patterns across algorithms (Table 3). The ethno-specific variant *CYP2C9* rs202201137 (A > G) demonstrated consistent overall influence (mean rank = 4.67), maintaining moderate importance across CART, GDB, and RFC. In contrast, *CYP2C9* rs2860905 (G > A) exhibited greater variability across models (mean rank = 6.0), contributing more prominently in CART and RFC than in GDBA. Among clinical variables, height in inches and BMI demonstrated strong but model-dependent importance (mean rank = 5.33 for both). Height ranked first in GDB and second in RFC, while BMI ranked first in CART and second in GDB, underscoring the continued relevance of non-genetic factors in warfarin dose variability. Age in years consistently ranked last across models (mean rank = 15), indicating minimal contribution within this dataset. For complex polygenic diseases, SNPs are currently considered the most informative features of genotype data [57,58]. To apply SNP information to machine learning, SNPs selected for predictive models were associated with loci contributing to drug metabolism [59]. We used permutation feature importance rankings to determine the impact of the SNPs related to low warfarin requirements in Puerto Ricans for warfarin sensitivity prediction (Table 3). Among additional genetic predictors, VKORC1*2, CYP4F2*3, and NQO1*2 demonstrated moderate contributions with algorithm specific variability. *CES2* rs4783745 (G > A) and CYP2C9*2 showed intermediate influence, whereas *ABCB1* rs10276036 (C > T) and *CYP2C9* Cluster ranked lower overall.

Collectively, these findings suggest that while ensemble methods provided stable predictive performance in small, admixed cohorts, feature importance may vary substantially across algorithms. Importantly, the consistent ranking of population-specific *CYP2C9* variants across models suggests that these loci may contribute to prediction in Puerto Rican cohorts, although the limited sensitivity observed indicated that additional genetic and clinical predictors were likely required.

Currently, routine pharmacogenetic testing for warfarin dosing is not universally implemented as the standard of care in Puerto Rico. While genotype-guided dosing is supported by the CPIC guidelines, testing has often been limited to research settings or selected cases of therapeutic instability. The development of population-specific predictive frameworks may support future integration of pharmacogenetics into routine anticoagulation management within Caribbean healthcare systems.

From a clinical perspective, the present findings should be interpreted cautiously. Although the models demonstrated moderate overall accuracy, the low sensitivity and positive predictive value observed across algorithms indicated limited ability to reliably identify warfarin-sensitive patients in this dataset. Consequently, these models should not be considered clinically actionable at this stage. Rather, the results demonstrate the feasibility of constructing predictive frameworks incorporating population-specific pharmacogenetic variants in admixed Caribbean cohorts, which will require larger datasets and external validation before clinical implementation can be considered. While current CPIC guidelines incorporate major variants such as *CYP2C9* and *VKORC1*, they may not fully capture variability in admixed Caribbean populations. The integration of ethno-specific variants such as rs202201137 into predictive algorithms warrants further investigation in larger cohorts to determine whether they contribute to improved dose stratification. However, the present model should be considered investigational, as prospective validation, cost effectiveness analyses, and integration into clinical decision support systems would be required before routine implementation in clinical practice.

This study has several important strengths. To our knowledge, it represents one of the first machine learning-based pharmacogenomic predictive frameworks developed specifically in Puerto Rican Caribbean Hispanics. The incorporation of ethno-specific *CYP2C9* variants that were rarely represented in European derived dosing models enhanced the population relevance of the approach. Additionally, we systematically compared multiple supervised learning algorithms under standardized validation procedures, allowing for robust internal model comparison. The use of permutation-based feature importance further strengthened interpretability by quantifying the relative contribution of both clinical and genetic predictors.

Several limitations should also be acknowledged. First, the effective modeling sample (*n* = 146) and small validation cohort (*n* = 52) limited statistical power, particularly for rare variant effects and minority class discrimination. The low sensitivity observed across the models likely reflected insufficient event counts rather than absence of biological signal. Second, class imbalance required oversampling within the training data, which may increase variance in model estimates despite the leakage of safe procedures. Third, this was a secondary analysis without external validation. Independent multi-center cohorts will be necessary to establish generalizability and clinical utility.

Future studies should prioritize the inclusion of larger and more diverse cohorts with harmonized clinical and pharmacogenomic data to improve feature distribution and reduce reliance on oversampling techniques. Standardized data collection protocols across studies will also be essential for enabling meta-analyses and validating novel genotype-phenotype associations relevant to warfarin dosing in underrepresented populations.

## 5. Conclusions

This study demonstrated the feasibility of applying machine learning approaches to pharmacogenetic prediction of warfarin sensitivity in Puerto Rican patients. While overall validation performance was modest and sensitivity for the minority class remained limited, ensemble tree-based methods provided the most stable discrimination. The consistent ranking of the ethno-specific *CYP2C9* variant rs202201137 supported further investigation of ancestry-informed pharmacogenetic refinement in Caribbean populations. Larger prospective studies with improved representation of warfarin-sensitive individuals will be required before clinical implementation can be considered.

## Figures and Tables

**Table 1:** Relevant characteristics of the Caribbean Hispanic patients.

Variables	Groups	
	Non-Sensitive Warfarin Dosing (%)	Sensitive Warfarin Dosing (%)
CYP2C9*2 (rs1799853; C > T)	119 (81.51)	27 (18.49)
C/C	85 (71.43)	17 (62.96)
C/T	32 (26.89)	8 (29.63)
T/T	2 (1.68)	2 (7.41)
*CYP2C9* rs202201137; c.1370A > G		
A/A	118 (99.16)	26 (96.30)
A/G	1 (0.84)	1 (3.70)
G/G	0 (0.00)	0 (0.00)
*CYP2C9* rs2860905 (G > A)		
G/G	59 (49.58)	9 (33.33)
G/A	51 (42.86)	14 (51.85)
A/A	9 (7.56)	4 (14.81)
*CYP2C9* rs1856908 (T > G)		
T/T	22 (18.49)	4 (14.81)
T/G	76 (63.87)	20 (74.07)
G/G	21 (17.65)	3 (11.11)
CYP2C9*8 (rs7900194; G > A)		
G/G	105 (88.24)	26 (96.30)
G/A	5 (4.20)	1 (3.70)
A/A	9 (7.56)	0 (0.00)
*CYP2C9* Cluster		
0	30 (25.21)	5 (18.52)
1	72 (60.50)	19 (70.37)
2	17 (14.29)	3 (11.11)
VKORC1*2 (rs9923231; G > A)		
G/G	55 (46.22)	11 (40.74)
G/A	52 (43.70)	13 (48.15)
A/A	12 (10.08)	3 (11.11)
NQO1*2 (rs1800566; C > T)		
C/C	86 (72.27)	24 (88.89)
C/T	26 (21.85)	2 (7.41)
T/T	7 (5.88)	1 (3.70)
CYP4F2*3 (rs2108622; c.1297G > A)		
G/G	93 (78.15)	20 (74.07)
G/A	20 (16.81)	6 (22.22)
A/A	6 (5.04)	1 (3.70)
*ABCB1* rs10276036 (C > T)		
C/C	20 (16.81)	3 (11.11)
C/T	91 (76.47)	23 (85.19)
T/T	8 (6.72)	1 (3.70)
*CES2* rs4783745 (G > A)		
G/G	101 (84.87)	25 (92.59)
G/A	16 (13.45)	2 (7.41)
A/A	2 (1.68)	0 (0.00)
Sex		
Male	95 (79.83)	23 (85.19)
Female	24 (20.17)	4 (14.81)
Age in Years (Mean ± SD)	65.59 ± 11.56	67.33 ± 14.79
Height in Inches (Mean ± SD)	66.53 ± 3.59	66.61 ± 2.38
BMI (Mean ± SD)	29.97 ± 6.33	29.71 ± 5.63

**Table 2:** Tuned warfarin classification performance of three best performing ML algorithms in validation set.

ML Algorithms	Accuracy	Precision	Recall (Sensitivity)	Specificity	PPV	NPV	PR-AUC	PR-AUC 95% CI
GDB	0.7500	0.7642	0.1429	0.8444	0.125	0.8636	0.1686	0.066–0.419
RFC	0.7308	0.7596	0.1429	0.8222	0.1111	0.8605	0.1786	0.068–0.442
CART	0.6538	0.7176	0	0.7556	0	0.8293	0.1346	0.057–0.231

**Table 3:** Permutation feature importance ranking for warfarin classification performance of ML algorithms.

Feature	Rankings CART	Rankings GDB	Rankings RFC	Rankings Mean
*CYP2C9* rs202201137 (A > G)	4	6	4	4.666667
VKORC1*2	4	5	6	5
BMI	1	2	13	5.333333
Height in Inches	13	1	2	5.333333
CYP4F2*3	4	6	7	5.666667
NQO1*2	4	12	1	5.666667
*CYP2C9* rs2860905 (G > A)	2	11	5	6
Sex	4	6	8	6
*CES2* rs4783745 (G > A)	4	13	3	6.666667
CYP2C9*2	3	4	14	7
*CYP2C9* rs1856908 (T > G)	4	6	11	7
CYP2C9*8	4	10	12	8.666667
*ABCB1* rs10276036 (C > T)	4	14	9	9
*CYP2C9* Cluster	14	3	10	9
Age in Years	15	15	15	15

## Data Availability

The raw data supporting the conclusions of this article will be made available by the authors on request.

## Abbreviations

The following abbreviations are used in this manuscript:

BMI	Body Mass Index
CART	Decision Tree Classifier
CPIC	Clinical Pharmacogenetics Implementation Consortium
CYP2C9	Cytochrome P450 Family 2 Subfamily C Member 9
CYP4F2	Cytochrome P450 Family 4 Subfamily F Member 2
GDB	Gradient Boosting Classifier
GWAS	Genome-Wide Association Studies
KNN	k-Nearest Neighbor
LDA	Linear Discriminant Analysis
LR	Logistic Regression
ML	Machine Learning
NB	Gaussian Naïve Bayes
NTI	Narrow Therapeutic Index
NQO1	NAD(P)H Quinone Oxidoreductase 1
PGx	Pharmacogenomics
PR-AUC	Area Under The Precision–Recall Curve
RFC	Random Forest Classifier
SMOTE	Synthetic Minority Oversampling
SNPs	Single Nucleotide Polymorphisms
SVM	Support Vector Machine Classifier
VKORC1	Vitamin K Epoxide Reductase Complex 1
